# Treatment-seeking behaviour and barriers to service access for sexually transmitted diseases among men who have sex with men in China: a multicentre cross-sectional survey

**DOI:** 10.1186/s40249-016-0219-5

**Published:** 2017-01-18

**Authors:** Jun-Jie Xu, Yan-Qiu Yu, Qing-Hai Hu, Hong-Jing Yan, Zhe Wang, Lin Lu, Ming-Hua Zhuang, Xi Chen, Ji-Hua Fu, Wei-Ming Tang, Wen-Qing Geng, Yong-Jun Jiang, Hong Shang

**Affiliations:** 1 Key Laboratory of AIDS Immunology of National Health and Family Planning Commission, Department of Laboratory Medicine, The First Affiliated Hospital, China Medical University, Shenyang, China; 2 Collaborative Innovation Center for Diagnosis and Treatment of Infectious Diseases, Hangzhou, China; 3 Jiangsu Provincial Centers for Disease Control and Prevention, Nanjing, China; 4 He’nan Provincial Centers for Disease Control and Prevention, Zhengzhou, China; 5 Yunnan Provincial Centers for Disease Control and Prevention, Kunming, China; 6 Shanghai Municipal Centers for Disease Control and Prevention, Shanghai, China; 7 Hu’nan Provincial Centers for Disease Control and Prevention, Changsha, China; 8 Shandong Provincial Centers for Disease Control and Prevention, Jinan, China; 9 University of North Carolina Project-China, Guangzhou, China

**Keywords:** Sexually transmitted diseases (STDs), Men who have sex with men (MSM), Treatment-seeking behaviour, HIV, China

## Abstract

**Background:**

Delayed or inappropriate treatment for sexually transmitted diseases (STDs) increases the risk of HIV acquisition and may cause other harmful outcomes. However, studies on STD treatment-seeking behaviour and correlated factors in men who have sex with men (MSM) are scarce.

This information is crucial for the promotion of STD treatment-seeking behaviour and reduction of HIV transmission among Chinese MSM.

**Methods:**

During 2012–2013, a multicentre cross-sectional study was conducted in 7 Chinese cities. Participants completed an interview-questionnaire and gave venous blood samples, which were then tested for antibodies to HIV, syphilis, and herpes simplex virus-2 (HSV-2). MSM who tested positive for syphilis/HSV-2 or had obvious STD-related symptoms within the last 12 months were defined as suspected STD-infected MSM.

**Results:**

Of the 4 496 eligible MSM who completed this survey, 24.4% (1 096/4 496) were categorized as suspected STD-infected MSM. 35.7% (391/1 096) of these MSM with suspected STD infections sought STD treatment in clinics within the last 12 months. Among MSM who did not attend STD clinics for treatment, the prevalence of syphilis and HSV-2 was significantly higher; the HIV prevalence and incidence within this subpopulation reached as high as 14.5% and 12.2/100 person-years, respectively. Multivariate logistic regression analysis indicated that having 7–12 years of education (vs. ≤6 years; a*OR*, 2.5; 95%*CI*, 1.0–6.1), ≥13 years of education (vs. ≤6 years: a*OR*, 2.8; 95%*CI*, 1.2–7.0), monthly income >500 USD (vs. ≤500 USD: a*OR*, 1.5; 95%*CI*, 1.1–2.1), obvious STD-related symptoms within last 12 months (a*OR*, 5.3; 95%*CI*, 3.7–7.5), being HIV infected (a*OR*, 1.7; 95%*CI*, 1.1–2.6), currently syphilis infected (a*OR*, 0.6; 95%*CI*, 0.4–0.9) and HSV-2 infected (a*OR*, 0.6; 95%*CI*, 0.5–0.9) were independent correlates with seeking STD treatment in clinics among Chinese MSM.

**Conclusions:**

The high prevalence of STD infection coupled with a low proportion of individuals who exhibit appropriate treatment-seeking behaviour create a high risk of a growing HIV epidemic among Chinese MSM. Models that prioritize better screening for and education about STDs should be urgently implemented, especially among low-income MSM.

**Electronic supplementary material:**

The online version of this article (doi:10.1186/s40249-016-0219-5) contains supplementary material, which is available to authorized users.

## Multilingual abstracts

Please see additional file 1 for translations of the abstract into the five official working languages of the United Nations.

## Background

Acquisition of other sexually transmitted diseases (STDs) may enhance susceptibility to HIV through a diverse array of biological mechanisms and vice versa [1]. Moreover, delayed or incorrect treatment of other STDs could hasten STD progression and further promote HIV transmission and acquisition [2]. As such, timely and improved treatment of other STDs can not only shorten disease duration but also reduce HIV incidence [3, 4]. Early STD detection and treatment may disrupt the chain of HIV/STD transmission and ought to be considered a key intervention strategy.

In order to achieve this goal of early intervention, key populations must be involved and engaged. Globally, men who have sex with men (MSM) compose an important high-risk population for HIV infection as well as STD infections [5], and China is no exception [6, 7]. Despite such high STD prevalence, however, behaviours related to STD treatment-seeking within this high-risk population have not been well investigated. Several studies have discussed the proportions of MSM who exhibit STD treatment-seeking behaviour for suspected STDs (South Africa, 49% [8]; Indonesia, 44.4% [9]; Hong Kong, 28.3% [10]; Chengdu, 81.1% [11]; Liuzhou, 87.0% [12]). However, very few of these studies investigated STD participants who did not know they were STD positive; in theory, the proportion of this subgroup of STD patients that exhibits STD treatment-seeking behaviour is small. Additionally, these past studies failed to analyse associated factors of STD treatment-seeking behaviour further, thus limiting their ability to provide evidence to aid in the development of effective STD treatment intervention strategies within this vulnerable population.

STD clinics in China can offer relatively high-quality diagnoses and treatment services for STD patients, but there exist several formidable obstacles to treatments. First, stigma hinders all aspects of STD testing, prevention and treatment [13]. STD treatment-seeking behaviour is still regarded as embarrassing and disgraceful for infected patients [14]. MSM experience additional stigma stemming from social condemnation of same-sex behaviour, prompting them to avoid seeking STD treatment in clinics [15]. These social perceptions interfere with STD prevention efforts directed toward at-risk populations [2] by implicitly discouraging treatment [14], thus aiding the further transmission of STDs. Instead, the convenient and private access to antibiotics in pharmacies has made self-medication the first option for many individuals who experience STD-related symptoms has made antibiotic misuse a serious concern in China [16]. This misuse of antibiotics may cause drug resistance, dysbacteriosis and toxic side effects [16], resulting in negative disease outcomes. Other obstacles to testing like inconvenient clinic hours and low educational levels among potential patients have been discussed in studies conducted in Vietnam [17], Laos [18], Tanzania [19], and China [13, 14, 20], etc. These studies, however, targeted general STD patients [13, 14, 20] and female sex workers (FSWs) [17–19], not MSM as a specific sub-category and, thus, it is not necessarily appropriate to apply these conclusions to the MSM population. Although the HIV testing behaviour of MSM has been studied extensively, the characteristics and factors associated with MSM treatment-seeking behaviour, especially as related to STD treatment-seeking in clinics, are barely known both domestically and internationally.

Based on the findings of past studies, we assumed that the prevalence of Chinese MSM who exhibited STD treatment-seeking behaviours in clinics was low. Past studies have also posited that this may interfere with the effects of HIV prevention and control programs targeting this vulnerable population. The current study aimed to understand characteristics of treatment-seeking behaviour among a geographically diverse group of Chinese MSM and to explore barriers to STD treatment-seeking in STD clinics in this high-risk population.

## Methods

### Study population and participant enrolment

Between June 2012 and June 2013, MSM were recruited through Internet sampling, venue-based sampling, and respondent-driven sampling (RDS) to participate in this cross-sectional study in 7 Chinese cities: Shenyang, Changsha, Kunming, Ji’nan, Nanjing, Shanghai and Zhengzhou. These 7 cities represent varying HIV prevalence; Shenyang and Ji’nan are low epidemic areas, Changsha, Nanjing and Shanghai are moderate epidemic areas, and Zhengzhou and Kunming are high epidemic areas. Individuals who met survey inclusion criteria: born biologically male, had anal sex with men within the last 12 months, were at least 18 years of age, and signed a written informed consent agreeing to participate – were enrolled in the study.

### Data collection

Standardized training was provided for all investigators, including volunteers and healthcare workers, prior to the commencement of the study. The same study protocol was implemented in the seven study cities. Investigators assigned each eligible MSM a unique personal identity number, and participants then completed anonymous questionnaires on socio-demographic characteristics, HIV-related knowledge, sexual history, recreational drug use, and healthcare-seeking behaviour.

The questionnaire’s socio-demographic characteristics questions included age, residential status, years of education, marital status, occupation, monthly income, and sexual orientation. Participants were asked eight questions on HIV-related knowledge, including HIV transmission routes and prevention strategies, and earned one point for each correctly answered question. Additional behavioural information was also collected, including main avenue for seeking male sexual partners, age of sexual debut with a male, the number of male sexual partners within last 6 months, history of condomless anal sex or commercial sex within last 6 months, history of recreational drug use (poppers, ecstasy, methamphetamine, amphetamine, tramadol, and ketamine) within last 6 months, and history of obvious STD-related symptoms and treatment-seeking behaviours within last 12 months.

Obvious STD-related symptoms were characterized as follows: 1) ever felt pain or burning sensation during urination, or had abnormal urethral discharge, or had traumatic abrasions or lumps and bumps on genitals or around anus, 2) ever had blisters on genitals, 3) ever had ulcers on genitals, or 4) ever had ulcers around anus.

### Laboratory testing

Participants provided 7 ml venous blood after pre-test counselling to test for HIV, syphilis, and HSV-2. Enzyme-linked immunosorbent assay (ELISA) (Vironostika HIV-1 Microelisa System; bioMerieux, Durham, NC) was performed to screen for HIV-1 antibody, and Western Blot (HIV Blot 2.2 WBTM, Genelabs Diagnostics, Singapore) was used to confirm positive cases. Syphilis was screened using a Rapid plasma reagin (RPR) test (Shanghai Kehua Bio-engineering Co., Ltd, China), and positive cases were confirmed by Treponema pallidum particle assay (TPPA) (Hainan Huamei, China). Only samples with positive results on both screening and confirmation tests were deemed HIV/syphilis positive. ELISA (HerpeSelect-2, Focus Technologies, USA) was used to determine the presence of an HSV-2 antibody infection. Confirmed HIV-positive specimens were tested for BED HIV-1 capture enzyme immunoassay (BED-CEIA, Calypte Biomedical Corporation, Rockville, MD, USA) to determine recently infected (HIV infected ≤168 days) or chronically infected (HIV infected >168 days) status [21].

### Data analysis

Double data entries and logic checks were performed using EpiData version 3.0 (The Epi Data Association Odense, Denmark). Suspected STD-infected MSM were defined as those who exhibited obvious STD-related symptoms within last 12 months or who tested positive on syphilis or/and HSV-2 tests. Self-reported treatment behaviour was split into two categories: seeking STD treatment in clinics (public STD clinics/ private hospitals) and failing to seek STD treatment in clinics (buying antibiotics in pharmacies without a prescription or seeking no treatment at all). Categorical variables were compared between MSM subgroups with different treatment-seeking behaviours using frequency and Chi-squared tests. HIV incidence was corrected for sensitivity of BED-CEIA assay using Hargrove correction. Incidence and its 95% confidence interval (95%*CI*) were calculated using the formula and latest adjusted parameters provided by China HIV reference test lab [22, 23]. Univariate logistic regression analysis was performed to determine Odds Ratios (*OR*) and 95%*CI*s for factors associated with seeking STD treatment in clinics among suspected STD-infected MSM subjects. Multivariate logistic regression analyses were conducted to estimate these associations once adjusted for potential confounding variables, including age, study city, residence status, marital status, and ethnicity in both models. Variables with two tailed *P* < 0.05 were retained in multivariate models. SAS version 9.2 (SAS Institute Inc., Cary, NC) was used for analysis.

### Ethic, consent and permissions

The study protocol and procedure were reviewed and approved by the Institutional Review Boards committee of the First Affiliated Hospital of China Medical University (2011 [24]). Details of the study were explained clearly for each participant, and written informed consent was obtained prior to the commencement of the survey.

## Results

### Demographic characteristics of total MSM participants

**Fig. 1:**
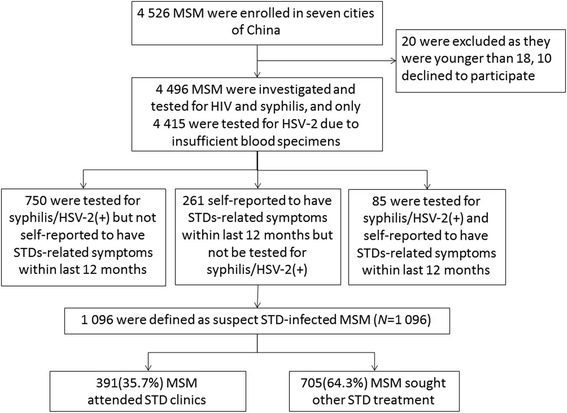
Flowchart of study of treatment-seeking behaviour among Chinese MSM

A total of 4496 eligible MSM were investigated in this study (Fig. 1). A majority of participants were 18–35 years of age (*n* = 3 483, 77.5%), never married (*n* = 3 135, 69.7%), and earned ≤500 USD per month (*n* = 2 809, 62.5%). Slightly more than half of the surveyed participants had completed, at least, college degrees (*n* = 2 379, 52.9%) and self-identified as homosexual (*n* = 2 659, 59.1%).

### Suspected STD infections

The prevalences of HIV, syphilis, and HSV-2 among all MSM participants were 9.9% (444/4 496), 8.5% (381/4 496), and 12.5% (552/4 415), respectively. 7.7% (346/4 496) of MSM self-reported at least one STD-related symptom within the last 12 months, among whom 79.8% (276/346) had mild STD-related symptoms like the feeling of pain or a burning sensation during urination, etc., 25.1% (87/346) had blisters on genitals, 11.9% (41/346) had ulcers on genitals, and 11.3% (39/346) had ulcers around anus. Based on the previously discussed study criteria, 1096 (24.4%) subjects were identified as suspected STD-infected MSM. Additionally, proportions of suspected STD infections varied geographically among the seven surveyed cities: 24.1% in Shenyang, 20.7% in Ji’nan, 30.3% in Changsha, 30.1% in Nanjing, 23.2% in Shanghai, 20.1% in Zhengzhou and 21.4% in Kunming, respectively.

### Socio-demographic characteristic and behaviours of suspected STD-infected MSM

Of the 1096 suspected STD-infected MSM, 35.7% sought STD treatment in clinics and 64.3% sought other STD treatment. Among all suspected STD-infected MSM, 20.3% (222/1 096) sought treatment in public hospitals, 25.5% (279/1 096) in public STD clinics and 2.6% (29/1 096) in private STD clinics while 8.1% (89/1 096) bought antibiotics in pharmacies without a prescription and 59.1% (648/1 096) sought no treatment at all. Along with significant variation in the prevalence of suspected STD infections among the seven surveyed cities, regional differences also existed in proportions of treatment-seeking behaviours: 12.5% sought STD treatment in clinics in Shenyang, 10.7% in Ji’nan, 49.3% in Changsha, 69.1% in Nanjing, 16.7% in Shanghai, 93.6% in Zhengzhou and 8.9% in Kunming, respectively.

Among suspected STD-infected MSM, a majority were 18–35 years of age (*n* = 785, 71.6%), never married (*n* = 707, 64.5%), earned ≤ 500 USD per month (*n* = 693, 63.2%) and sought male sexual partners mainly via the Internet (*n* = 696, 63.5%). More than half correctly answered all HIV-related questions (*n* = 629, 57.4%) and self-identified as homosexual (*n* = 650, 59.3%). 29.5% of this population self-identified as bisexual, likely as a consequence of the severe stigma associated with homosexuality and pressure to produce offspring in China; within this bisexual population, there exists a high risk of HIV/STD transmission to general population as a consequence of incomplete sexual behaviour disclosure.

Socio-demographic characteristics and HIV risk behaviours were compared between MSM who sought STD treatment in clinics and those who did not seek STD treatment in clinics. MSM who sought treatment in clinics were more educated (55.2% ≥ 13 years vs. 44.7%; *P* < 0.01) and fewer had engaged in anal sex with male sexual partners within last 6 months (91.6% vs. 96.5%; *P* < 0.01). Other characteristics were not significantly statistically different between these 2 MSM subgroups (each *P* > 0.05).

**Fig. 2:**
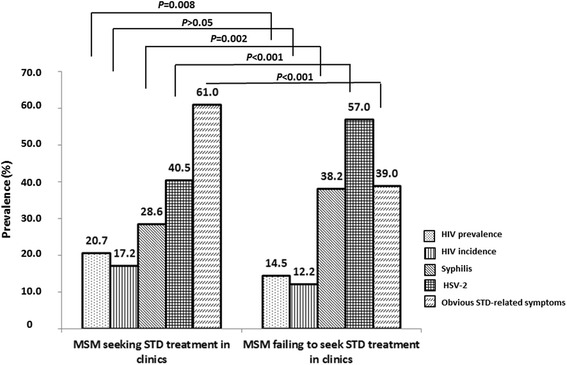
HIV/suspected STD prevalence of Chinese MSM. Figure 2 shows HIV prevalence, HIV incidence, and prevalence of Syphilis, HSV-2 and obvious STD-related symptoms among MSM seeking STD treatment in clinics and the rest MSM failing to attend STD clinics in seven cities of China

We also compared presentation of obvious STD-related symptoms and STD test results between the two treatment-seeking MSM subgroups. MSM subjects who sought STD treatment in clinics had a statistically lower prevalence of detected STDs compared to those who did not, including syphilis (28.6% vs. 38.2%; *P* < 0.01) and HSV-2 (40.5% vs. 57.0%; *P* < 0.01), and fewer mild, obvious STD-related symptoms (73.9% vs. 88.9%; *P* < 0.01). However, HIV prevalence (20.7% vs. 14.5%; *P* = 0.01) of this MSM subgroup was significantly higher than those who did not attend STD clinics. Among 183 HIV-positive MSM, 69 had recently acquired HIV infections, while 179 had chronic infections, detected using BED-CEIA. The calculated crude HIV incidences for MSM who sought STD treatment in clinics and those who did not were 17.7 (95%*CI*, 11.4–24.1)/100 person-years (PYs) and 12.5 (95%*CI*, 8.6–16.5)/100PYs, respectively. The calculated Hargrove-adjusted HIV incidences were 17.2 (95%*CI*, 11.0–23.3)/100PYs and 12.2 (95%*CI*, 8.4–16.1)/100PYs, respectively (Fig. 2).

### Factors associated with STD treatment-seeking in clinics among suspected STD-infected MSM

Table 1 presents the univariate and multivariate logistic regression analysis of factors associated with STD treatment-seeking in clinics among suspected STD-infected MSM in 7 Chinese cities. In multivariate analysis, having 7–12 years of education (vs. ≤6 years; a*OR*, 2.5; 95%*CI*, 1.0–6.1), ≥13 years of education (vs. ≤6 years: a*OR*, 2.8; 95%*CI*, 1.2–7.0), monthly income ≤500USD (vs. >500 USD: a*OR*, 0.7; 95%CI, 0.5–0.9), having obvious STD-related symptoms within last 12 months (a*OR*, 5.3; 95%*CI*, 3.7–7.5), and being HIV infected (a*OR*, 1.7; 95%*CI*, 1.1–2.6), currently syphilis infected (a*OR*, 0.6; 95%*CI*, 0.4–0.9) and HSV-2 infected (a*OR*, 0.6; 95%*CI*, 0.5–0.9) were found to be significantly associated with seeking STD treatment in hospitals/clinics (each *P* < 0.05).

**Table 1:** Factors associated with STD treatment to clinics among suspected STD-infected MSM

Variables	*N*	MSM attending STD treatment to clinics		Crude model	Adjusted model
		*N*	%	*OR* (95%*CI*)	a*OR* (95%*CI*)
overall	1 096	391	35.7		
Length of education (years)					
≤6	57	10	17.5	ref	ref
7–12	508	165	32.5	2.3 (1.1–4.6)*	2.5 (1.0–6.1)*
≥ 13	531	216	40.7	3.2 (1.6–6.5)**	2.8 (1.2–7.0)*
Sexual orientation					
Other orientation	446	155	34.8	ref	ref
Homosexual orientation	650	236	36.3	1.1 (0.8–1.4)	0.8 (0.6–1.1)
Occupation					
Others	728	256	35.2	ref	ref
Students	98	39	39.8	1.2 (0.8-1.9)	1.0 (0.5-1.7)
Business service providers	270	96	35.6	1.0 (0.8–1.4)	1.1 (0.7–1.7)
Income (USD)					
≤500	693	242	34.9	ref	ref
>500	403	149	36.9	1.1 (0.8–1.4)	1.5 (1.1–2.1)*
Main venue of seeking male sexual partners					
Non-internet	400	134	33.5	ref	ref
Internet	696	257	36.9	1.2 (0.9–1.5)	1.3 (0.9–1.9)
HIV knowledge questions					
All correct	629	224	35.6	ref	ref
Partly correct	467	167	35.8	1.0 (0.8–1.29)	1.1 (0.8–1.6)
Used condom at last anal sex with male sexual partners					
Yes	756	276	36.5	ref	ref
No	340	115	33.8	1.1 (0.9–1.5)	1.1 (0.8–1.5)
No. of male sexual partners in last 6 months					
≤2	619	231	37.3	ref	ref
>2	477	160	33.5	0.9 (0.7–1.1)	0.9 (0.6–1.2)
Ever bought sex in last 6 months					
No	1 019	369	36.2	ref	ref
Yes	77	22	28.6	0.7 (0.4–1.2)	1.3 (0.7–2.3)
Ever sold sex in last 6 months					
No	1 001	362	36.2	ref	ref
Yes	95	29	30.5	0.8 (0.5–1.2)	1.6 (0.9–2.8)
Obvious STD-related symptoms in last 12 months					
No	750	180	24.0	ref	ref
Yes	346	211	61.0	5.0 (3.8–6.5)**	5.3 (3.7–7.5)**
HIV					
Negative	913	310	34.0	ref	ref
Positive	183	81	44.3	1.6 (1.1–2.1)**	1.7 (1.1–2.6)*
Syphilis					
Negative	715	279	39.0	ref	ref
Positive	381	112	29.4	0.7 (0.5–0.9)**	0.6 (0.4–0.9)**
HSV-2					
Negative	528	229	43.4	ref	Ref
Positive	552	156	28.3	0.5(0.4–0.7)**	0.5(0.4–0.8)**

Note: 16 MSM failed to test for HSV-2 antibody for lack of sufficient blood specimens; *MSM*: men who have sex with men; *STD* sexually transmitted disease, *HSV*-2 herpes simplex virus-2, a*OR* adjusted odds ratio, *CI* confidence interval; ref: reference group #

Multivariate models were adjusted for age, study city, residence, marital status and ethnicity

*P < 0.05, **P < 0.01

## Discussion

To the best of our knowledge, this is the first multisite study that explores factors related to STD treatment-seeking behaviour among MSM in China. Our study expands upon existing knowledge of the reasons for the severe HIV/AIDS epidemic among Chinese MSM. Improving patterns of STD treatment-seeking behaviour and reducing HIV transmission among MSM requires understanding this information. Our results can also be used as a reference for researchers who wish to carry out in-depth studies on STD treatment and measures among MSM populations, both within China and internationally.

The prevalence of suspected STD infections (24.4%) found in our study was much higher than reported among MSM in Guangzhou (23.2% in 2012 and 17.0% in 2013) [25] and central Brazil (4.3%) [26], but lower than among MSM in the Jiangsu province of China (34.1%) [27] and El Salvador (49.8%) [28] and among female sex workers (FSWs) in Shanxi province (53.6%) [29] of China (the comparisons of prevalence of suspected STD infection were all made under the same definition). The HIV prevalence among suspected STD-infected MSM was 16.7%, which was also higher than Chinese MSM in general (6.5%) [30] and Chinese FSWs (0.2%) [31], as reported in two recently published meta-analyses. This indicates that STD clinics and medical institutions at all levels ought to recommend HIV screening for suspected STD-infected MSM through provider-initiated HIV testing and counselling (PITC). This could aid in early discovery of HIV infections, thus laying the foundation for timely HIV treatment and behavioural interventions.

Alarmingly, slightly more than one-third (35.7%) of suspected STD-infected MSM in our survey sought STD treatment in clinics; although this was slightly higher than reported data about MSM in Hong Kong [10], it is lower than South Africa [8] and Indonesia [9], and almost 2 to 3 times lower than Liuzhou (87.0%) [12] and Chengdu (81.1%) [11]. The discrepancy in participants’ STD treatment-seeking behaviour may be partly attributable to the differences in survey subject recruitment standards. Our study also included participants who did not know their STD testing statuses, while previous studies did not; it is believed that a small proportion of men in this subgroup exhibit STD treatment-seeking behaviour. Concurrently with the ongoing goal of “Ending the HIV epidemic by 2030” proposed by the Joint United Nations Programme on HIV and AIDS (UNAIDS), the World Health Organization (WHO) also proposes the target of ending STD epidemics as public health concerns. This relates directly to the continuum of available STD service, including accessibility of STD testing and access to short-term care, treatment and chronic care [32]. However, the low levels of STD treatment in our study indicate that current STD prevention and control efforts in China are far from sufficient. Additionally, this study found that MSM who failed to seek STD treatment in clinics had a significantly higher prevalence of syphilis/HSV-2 than those who sought STD treatment in clinics. This poses a non-trivial threat to the control of HIV transmission and, to a certain extent, explains high HIV incidence [33] and the rising HIV prevalence among China’s MSM population [6]. This may be attributable to the distinct objectives of HIV and STD prevention and control efforts in China. The National Center for AIDS Prevention and Control (NCAIDS) of China Centers for Disease Control (CDC) system focuses more heavily on HIV-related work, while the National Center for Sexually Transmitted Disease (NCSTD) of China CDC focuses on STDs. Integrated HIV and sexual reproductive health (SRH) services could reduce high-risk sexual behaviours and improve the utilization of SRH services [34]. Expanding physician training on STD treatment and management may be a similarly efficient strategy in preventing HIV/STD transmission [35]. Therefore, as a response to WHO’s goal, Chinese policymakers and healthcare workers in CDCs ought to adopt the aforementioned measures and work to establish long-term cooperation between public health institutions and local medical services through policies like efficient transfer protocols for STD-infected MSM to formal hospitals. By adopting such an approach, with the objective of promoting their STD treatment-seeking behaviour and curing STDs as soon as possible after diagnosis of infection, health professionals can further decrease the risk of MSM transmitting and acquiring HIV.

This study also investigated barriers to STD treatment-seeking in clinics among MSM in order to offer evidence about ways in which STD treatment-seeking behaviour might be improved and HIV transmission risk reduced. Suspected STD-infected MSM with higher incomes were more likely to attend STD clinics, consistent with results from studies in Nigeria [36] and several Chinese cities [13, 14, 37]. Lower incomes was associated with poorer choices of treatment for STDs, for several reasons, including its correlation with lower educational level as well as unaffordable STD services and a general lack of appropriate healthcare resources in certain communities [24]. Additionally, easy access to antibiotics in pharmacies acts functions as a good alternative to time-consuming, potentially costly clinic visits. As such, the government ought to launch economic assistance or compensation initiatives for essential STD treatments in serious HIV epidemic areas. This observation similarly underscores the need for greater regulation of the sale and purchase of antibiotics in pharmacies.

Suspected STD-infected subjects with higher education levels were also more likely to seek STD treatment in clinics, which corroborated previous studies [13, 14, 36, 37]. It indicates subjects with better education could be more knowledgeable about STDs and had more access to necessary STD education, resulting to their higher rates of clinic-based treatment seeking behaviour. In contrast, regarding those with lower education level who tended to underestimate their STD symptoms due to insufficient STD-related knowledge, simple and direct measures like videos or posters could be utilized to popularize information about STD prevention and treatment among them.

This study also found that MSM infected with syphilis and HSV-2 were less likely to seek STD treatment in clinics, which is an interesting but alarming phenomenon. One possible explanation is that symptoms of these two STDs are easily neglected in their early infection stage. Additionally, social stigma deters individuals from seeking STD treatment in clinics, as STDs are still considered shameful and are viewed as “losing face” China. In order to address this issue, it is imperative that public health sectors scale up STD testing and education targeted at the MSM population.

MSM who experienced obvious STD-related symptoms were more likely to seek STD treatment in clinics, according to this study. However, this may only be the case for certain noticeable symptoms, as we also observed that MSM who experienced mild STD symptoms tended to buy antibiotics in pharmacies or sought no treatment at all. For this reason, public health institutions should not only perform tests for STDs that have noticeable symptoms like syphilis or HSV-2, but should also strengthen and expand the screening for asymptomatic STDs among MSM that can have serious consequences in their later stages, like Chlamydia and gonorrhoea. More thorough testing would help this high-risk population to understand STD infection statuses more completely, allowing them to make fully informed, correct choices about treatment.

Among MSM subgroup who sought STD treatment in clinics, HIV prevalence was significantly higher than those who did not, which is a surprisingly, seemingly contradictory phenomenon that is, nevertheless, consistent with a similar study conducted in Germany [15]. A possible explanation is public hospitals or clinics are usually equipped with professional HIV diagnosis facilities [38], so suspected STD-infected MSM who sought STD treatment in clinics may have high chance of being screened for HIV, therefore had higher prevalence.

Although the HIV prevalence and incidence in the subgroup of MSM who did not seek STD treatment in clinics are relatively lower, it still reached up to 14.5% and 12.2/100PYs, respectively. The epidemic synergistic effect of STDs on HIV acquisition risk means that these figures may continue to increase within this subpopulation. Public health authorities should pay more attention to interventions aimed to address treatment-seeking behaviours among MSM and ought to consider them a crucial part of any HIV epidemic prevention measures in China.

This study’s strengths include its large sample size and the different levels of HIV epidemic zones in China represented by the seven investigated cities. All study sites utilized a uniform study protocol, and carefully prescribed training was conducted for all investigators and healthcare workers to ensure the quality and consistency of the study. Inevitably, however, there remain some limitations. Typical STD symptoms and standard laboratory tests for syphilis and HSV-2 were used as standards to determine whether subjects were recently infected with STDs. These standards do not include all STD symptoms and STD items that could be tested in a laboratory; to some extent, this approach may underestimate the prevalence of STD infections. As such, caution should be used when explaining or extrapolating the study’s results. Moreover, owing to the nature of cross-sectional studies, causal inferences could not be made, and social desirability could exist. As such, we interpreted results with caution. Although we adjusted for possible confounding variables in multivariate models, it is possible that some confounders were omitted.

## Conclusions

The prevalence of suspected STD infection among MSM in this multisite study was alarmingly high. Only slightly more than one-third of MSM with suspected STD infections sought treatment in clinics, suggesting a high risk of HIV acquisition and transmission. These phenomena should not be ignored. The public health sector should work with local medical institutions to maximize the efficiency of HIV/STD prevention and control programs. Targeted interventions of STD treatment improvement should focus especially on MSM with low income and less education. Additionally, screening for asymptomatic STDs ought to be strengthened and expanded.

## Additional file

Additional file 1: Multilingual abstracts in the five official working languages of the United Nations. (PDF 645 kb)

## Abbreviations

BED-CEIA: BED HIV-1 capture enzyme immunoassay
ELISA: Enzyme-linked immunosorbent assay
FSW: Female sex worker
HSV-2: Herpes simplex virus-2
MSM: Men who have sex with men
NCAIDS: National center for AIDS Prevention and Control
NCSTD: National center for STD prevention and Control
OR: Odds ratio
PITC: Provider-initiated HIV testing and counselling
RDS: Respondent-driven sampling
RPR: Rapid plasma regain
SRH: Sexual reproductive health
STD: Sexually transmitted disease
TPPA: Treponema pallidum particle assay
UNAIDS: The joint United Nation programme on HIV and AIDS
