# Supplementary material for: Treatment-seeking behaviour and barriers to service access for sexually transmitted diseases among men who have sex with men in China: a multicentre cross-sectional survey
Source: Infect Dis Poverty. 2017 Jan 18;6:15. doi: 10.1186/s40249-016-0219-5 (PMC5242014; doi:10.1186/s40249-016-0219-5)
Supplement: Additional file 1: — Multilingual abstracts in the five official working languages of the United Nations. (PDF 645 kb) [file 40249_2016_219_MOESM1_ESM.pdf]

Translation of the abstract into the five official working languages of the United Nations

سلوك السعي إلى العلاج وعوائق الحصول على الخدمات الصحية المتعلقة بمعالجة الأمراض المنقولة جنسيًا بين الرجال الذين يمارسون الجنس مع الرجال (MSM) في الصين: دراسة استقصائية مقطعية متعددة المراكز

Jun-Jie Xu, Yan-Qiu Yu, Qing-Hai Hu, Hong-Jing Yan, Zhe Wang, Lin Lu, Ming-Hua Zhuang, Xi Chen, Ji-Hua Fu, Wei-Ming Tang, Wen-Qing Geng, Yong-Jun Jiang, Hong Shang

الملخص

نبذة طبية: إن التأخر في معالجة الأمراض المنقولة جنسيًا (STDs) أو معالجتها بشكل غير صحيح يزيد من مخاطر الإصابة بفيروس نقص المناعة البشرية (HIV)، وقد ينتج عن ذلك أيضًا تبعات أخرى ضارة. ومع ذلك، فإن الدراسات المعنية بسلوك السعي إلى طلب العلاج من الأمراض المنقولة جنسيًا (STDs) بين الرجال الذين يمارسون الجنس مع الرجال (MSM) والعوامل المرتبطة به قليلة جدًا. يُعد توافر مثل هذه المعلومات عامل أساسي من أجل تعزيز سلوك السعي إلى طلب العلاج من الأمراض المنقولة جنسيًا (STDs) والحد من انتقال فيروس نقص المناعة البشرية (HIV) بين الرجال الذين يمارسون الجنس مع الرجال (MSM) في الصين.

طرق القياس: خلال عامي 2012 و2013 أجريت دراسة استقصائية مقطعية متعددة المراكز في سبع مدن صينية. خلال المقابلات، أجاب المشاركون على أسئلة الاستبيان، وأخذت منهم عينات دم عن طريق الوريد. ثم أجريت على هذه العينات اختبارات البحث عن الأجسام المضادة لفيروس نقص المناعة البشرية (HIV)، والزهري، وفيروس الهربس البسيط من النوع 2 (HSV-2). الرجال الذين يمارسون الجنس مع الرجال (MSM) الذين ثبت إصابتهم بمرض الزهري أو فيروس الهربس البسيط من النوع 2 (HSV-2) أو كانت لديهم خلال ال 12 شهرًا الأخيرة أعراضًا واضحة تتعلق بأمراض منقولة جنسيًا (STDs) تم تحديدهم ك "رجال يمارسون الجنس مع الرجال (MSM) مُشتبه بإصابتهم بأمراض منقولة جنسيًا (STDs)".

النتائج: من بين 4496 رجل يمارسون الجنس مع الرجال (MSM) شاركوا في هذه الدراسة الاستقصائية، تم تصنيف 24.4% منهم (أي: 1096 رجل من ال 4496 رجل) ك "رجال يمارسون الجنس مع الرجال (MSM) مُشتبه بإصابتهم بأمراض منقولة جنسيًا (STDs)". 35.7% من هؤلاء الرجال (أي:391 رجل من ال 1096 رجل) الذين يمارسون الجنس مع الرجال (MSM) ويُشتبه في إصابتهم بأمراض منقولة جنسيًا (STDs) سعوا خلال ال 12 شهرًا الأخيرة إلى العلاج من المرض المنقول جنسيًا (STD) لدى عيادات معالجة الأمراض المنقولة جنسيًا. هذا وقد كان معدل انتشار مرض الزهري وفيروس الهربس البسيط من النوع 2 (HSV-2) أعلى بشكل ملحوظ بين الشريحة من الرجال الذين يمارسون الجنس مع الرجال (MSM) الذين لم يذهبوا إلى عيادات معالجة الأمراض المنقولة جنسيًا للعلاج. بلغ معدل انتشار فيروس نقص المناعة البشرية (HIV) 14.5% في هذه الشريحة السكانية، وبلغ معدل الإصابة بالمرض بينهم 12.2 شخص من بين كل 100 شخص في السنة. وقد أوضح تحليل الانحدار اللوجستي متعدد المتغيرات أنه من بين الرجال الذين يمارسون الجنس مع الرجال (MSM) في الصين كان الأشخاص الذين تلقوا تعليمًا لمدة ما بين 7 سنوات إلى 12 سنة (مقابل الأشخاص الذين تلقوا تعليمًا لمدة ≥ 6 سنوات؛ a*OR*، 2.5؛ 95٪ CI، 6.1-1.0)، والأشخاص الذين تلقوا تعليمًا لمدة ≤ 13 سنة (مقابل الأشخاص الذين تلقوا تعليمًا لمدة ≥ 6 سنوات؛ a*OR*، 2.8؛95٪ CI، 1.2-7.0)، والأشخاص الذين يبلغ دخلهم الشهري > 500 دولار أمريكي (مقابل الأشخاص الذين يبلغ دخلهم الشهري ≥ 500 دولار أمريكي؛ aOR، 1.5؛95٪ CI، 2.1-1.1)، والأشخاص الذين كانت لديهم خلال ال 12 شهرًا الأخيرة أعراضًا واضحة تتعلق بمرض منقول جنسيًا (STD) (aOR، 5.3؛95٪ CI، 7.5-3.7)، والأشخاص المصابون بفيروس نقص المناعة البشرية (HIV) (aOR، 1.7؛95٪ CI، 2.6-1.1)، والأشخاص المصابون حاليًا بالزهري (aOR، 0.6؛95٪ CI، 0.9-0.4)، والأشخاص المصابون بفيروس الهربس البسيط من النوع 2 (HSV-2) (aOR، 0.6؛95٪ CI، 0.9-0.5) مرتبطين بشكل مستقل بالسعي إلى طلب العلاج من الأمراض المنقولة جنسيًا (STDs) لدى عيادات معالجة الأمراض المنقولة جنسيًا.

الاستنتاجات: إن ارتفاع معدل انتشار الإصابة بالأمراض المنقولة جنسيًا (STDs)، في ظل قلة عدد من يظهرون سلوك السعي إلى طلب العلاج من الأمراض المنقولة جنسيًا (STDs)، يخلق مخاطرًا عالية بشأن تزايد انتشار وباء فيروس نقص المناعة البشرية (HIV) بين الرجال الذين يمارسون الجنس مع الرجال (MSM) في الصين. النماذج التي تعطي الأولية لإجراء مسح أفضل حول الأمراض المنقولة جنسيًا (STDs) ونشر الوعي بشأنها ينبغي أن تنفذ على وجه السرعة، وخاصة بين الشريحة السكانية من "الرجال الذين يمارسون الجنس مع الرجال (MSM)" ذوي الدخل المنخفض.

Translated from English version into Arabic by Ahmed Gad Elrab, through

TRANSLATORS WITHOUT BORDERS

中国男男性接触者性病求医行为及其影响因素的多中心横断面调查

Jun-Jie Xu, Yan-Qiu Yu, Qing-Hai Hu, Hong-Jing Yan, Zhe Wang, Lin Lu, Ming-Hua Zhuang, Xi Chen, Ji-Hua Fu, Wei-Ming Tang, Wen-Qing Geng, Yong-Jun Jiang, Hong Shang

摘要

背景：性传播疾病（sexually transmitted diseases，STDs）的延误治疗或不规范治疗行为会加速患者的疾病进展，并增加其性病/HIV 的传播和感染风险。因此，了解我国男男性接触者（men who have sex with men, MSM）人群的性病求医行为特征及其影响因素，对于提高该人群的性病求医行为和降低 HIV 传播风险具有至关重要的意义。然而，现国内外缺乏针对 MSM 人群求医行为的系统研究。

方法： 2012 年 6 月至 2013 年 6 月本项目在中国 7 市(北京、上海、南京、沈阳、长沙、济南、昆明)招募 MSM 人群开展了横断面调查。对每位符合入选条件的调查对象进行访谈式问卷调查，同时采集血样进行 HIV、梅毒和 II 型单纯疱疹病毒（HSV-2）抗体检测。将梅毒和/或HSV-2抗体阳性者或过去12个月自报出现典型性病症状者定义为疑似性病感染MSM。

结果：本研究共纳入 4496 名 MSM，其中 24.4%定义为疑似性病感染 MSM。35.7%(394/1096)的疑似性病感染 MSM 在过去 12 个月内曾到医院(公立性病门诊/私人门诊)寻求性病治疗。未到医院寻求治疗的疑似性病感染 MSM 人群的梅毒和 HSV-2 感染率均较治疗者高，且其 HIV 感染率和新发感染率分别高达 14.5%和 12.2/100 人年。多因素 logistic 回归分析结果显示，接受过 7-12 年学校教育（vs. ≤6 年学校教育; a*OR*, 2.5; 95% CI, 1.0-6.1），接受过≥13 年学校教育（vs. ≤6 年学校教育: a*OR*, 2.8; 95% CI, 1.2-7.0）, 月收入>500 美元（vs. ≤500 美元: a*OR*, 1.5; 95% CI, 1.1-2.1），过去 12 个月内有明显性病症状（a*OR*, 5.3; 95% CI, 3.7-7.5），感染 HIV（a*OR*, 1.7; 95% CI, 1.1-2.6），现患梅毒（aOR,0.6; 95% CI, 0.4-0.9）和感染 HSV-2（a*OR*, 0.6; 95% *CI*, 0.5-0.9）是我国 MSM 人群去医院寻求性病治疗的独立影响因素。

结论：我国 7 省市 MSM 人群性病感染率高，且寻求规范性病求医行为比例低，这一现象可加快该人群的 HIV 传播和感染风险，并进一步扩散我国的 HIV 疫情。因此相关卫生部门亟需进一步加强我国 MSM 人群的性病宣传教育和性病筛查规模，尤其是针对低收入 MSM 亚人群进行重点干预。

Translated from English version into Chinese by Jun-Jie Xu, Yan-Qiu Yu

Comportement de recherche de traitement et obstacles à l'accès aux services de traitement de maladies sexuellement transmissibles parmi les hommes ayant des rapports homosexuels en Chine : une étude transversale

Jun-Jie Xu, Yan-Qiu Yu, Qing-Hai Hu, Hong-Jing Yan, Zhe Wang, Lin Lu, Ming-Hua Zhuang, Xi Chen, Ji-Hua Fu, Wei-Ming Tang, Wen-Qing Geng, Yong-Jun Jiang, Hong Shang

Résumé

Contexte : l'obtention d'un traitement retardé ou inapproprié de maladies sexuellement transmissibles (MST) augmente le risque d'infection par le VIH et peut causer d'autres résultats néfastes. Néanmoins, les études menées sur le comportement de recherche de traitement de MST et les facteurs corrélés chez des hommes ayant des rapports homosexuels sont insuffisantes. Ces informations sont cruciales pour la promotion du comportement de recherche de traitement de MST et la réduction de la transmission du VIH parmi les hommes chinois ayant des rapports homosexuels.

Méthodes : une étude transversale multicentrique a été menée dans sept villes chinoises de 2012 à 2013. Les participants ont rempli un questionnaire et ont donné des échantillons de sang veineux ayant ensuite fait l’objet d’un dépistage d’anticorps contre le VIH, la syphilis et le virus herpès simplex (VHS-2). Les hommes ayant des rapports homosexuels recevant un résultat positif suite au dépistage de la syphilis/VHS-2 ou présentant des symptômes manifestes liés à des MST au cours des 12 derniers mois ont été définis comme des hommes ayant des rapports homosexuels chez lesquels on suspecte une infection par des MST.
Résultats : sur les 4496 hommes ayant des rapports homosexuels éligibles au cours de cette étude, 24,4 % (1096/4496) ont été classés comme étant des hommes ayant des rapports homosexuels chez lesquels on suspecte une infection par des MST. 35,7 % (391/1096) de ces hommes ayant des rapports homosexuels chez lesquels on suspecte une infection par des MST ont recherché un traitement anti-MST au sein d’établissements de soins de santé au cours des 12 derniers mois. La prévalence de la syphilis et du VHS-2 était significativement élevée parmi les hommes ayant des rapports homosexuels qui ne se sont pas rendus auprès d’établissements de prise en charge des MST afin d’y recevoir un traitement. La prévalence et l’incidence du VIH au sein de cette sous-population ont atteint respectivement 14,5 % et 12,2/100 journées-personnes. L’analyse de régression logistique multivariée a indiqué que le fait d’avoir été scolarisé pendant 7 à 12 ans (vs. ≤6 ans ; a*OR*, 2,5 ; *IC* à 95 %, 1,0-6,1), ≥13 ans de scolarisation (vs. ≤6 ans , a*OR*, 2,8 ; *IC* à 95 %, 1,2-7,0), d’afficher un revenu mensuel >500 dollars US (vs. ≤500 dollars US : a*OR*, 1,5 ; *IC* á 95 %, 1,1-2,1), d’avoir présenté des symptômes manifestes liés à des MST au cours des 12 derniers mois (a*OR*, 5,3 ; *IC* à 95 %, 3,7-7,5), d’être infecté par le VIH (a*OR*, 1,7 ; *IC* à 95 %, 1,1-2,6), d’ être actuellement infecté par la syphilis (a*OR*, 0,6 ; *IC* à 95 %, 0,4-0,9) et d’être infecté par le VHS-2 (a*OR*, 0,6 ; *IC* à 95 %, 0,5-0,9) constituaient des corrélations indépendantes par rapport à la recherche de traitement anti-MST au sein d’établissements de soins de santé parmi les hommes chinois ayant des rapports homosexuels.
Conclusions : la forte prévalence de l’infection par des MST associée à la faible proportion d’individus présentant un comportement de recherche de traitement approprié engendrent un risque élevé de croissance de l’épidémie de VIH parmi les hommes chinois ayant des rapports homosexuels. Des modèles axés sur le meilleur dépistage des MST et sur la sensibilisation à leur égard doivent impérativement être mis en œuvre, notamment parmi les hommes ayant des rapports homosexuels à faible revenu.

Translated from English version into French by eric ragu, through

TRANSLATORS
WITHOUT BORDERS

Поведение лечение-поиска и барьеры для доступа к услугам ЗППП на половом поведении между мужчинами в Китае: Одно много-центровое исследование.

Джун-Дже Щу, Йан-Чью Юй, Чин-Хай Ху, Хун-Тин Йан, Дже Ван, Лин Лу, Мин-Хуа Чан, Син Чен, Джи-Хуа Фу, Вий-Мин Тан, Вен-Чин Ген, Йон-Джун Джиан, Хун Шан.

Реферат

Фон: Несвоевременное или неподходящее лечение к ЗППП повышает риск СПИД-инфекцией и причиняет серьёзный вред здоровью. Однако, исследование о ЗППП поведение лечение-поиска и коррелированный фактор о половом поведении между мужчинами-это

скудный редкие. Это информация очень важная для продвижения ЗППП поведение лечение-поиска и редукции инфекции СПИД между мужчинами в Китае.
Методы: В 2012-2013 году, одно много-центровое междисциплинарное исследование приводилось в семи городах Китая. Участники устроили анкетировании и дали венозные образцы крови, которые были протестированные на антивещество к ВИЧ, сифилису и ВПГ-2. МСМ (Мужчины, практикующие секс с мужчинами), в сифилисе/ВПГ-2 антитела положительные или имеют очевидные ЗППП-родственные симптомы в прошлое течение 12 месяцев определяются как подозреваемый ЗППП-инфекции МСМ.
Результатs: Среди 4496 желательных МСМ, которые сделали этот опрос, 24.5%(1096/4496) были классифицированы в качестве подозреваемом ЗППП-инфекции МСМ. 35.7% (391/1096) среди этих МСМ с подозреваемым ЗППП-инфекцией ищут лечение ЗППП в клиниках в прошлое течение 12 месяцев. Посреди МСМ, которые не принимают лечение в клиниках, распространённость сифилиса и ВПГ-2 были значительно выше. Распространённость ВИЧ и охват в субпопуляции достигали 14.5% и 12.2/100 лиц-годы в указанном порядке. Многомерный анализ логистической регрессии проявил что, у которых 7~12 лет образования (против ≤6 лет; aOR, 2.5; 95%CI, 1.0-6.1), ≥13 лет образования (против. ≤6 years: aOR, 2.8; 95%CI, 1.2-7.0), месячный доход >500 американских долларов (против ≤500 американских долларов: aOR, 1.5; 95%CI, 1.1-2.1), очевидные ЗППП-родственные симптомы в прошлое течение 12 месяцев (aOR, 5.3; 95%CI, 3.7-7.5), ВИЧ-инфекции (aOR, 1.7; 95%CI, 1.1-2.6), сифилис инфицированного в настоящее время (aOR, 0.6; 95%CI, 0.4-0.9) и ВПГ-2 инфицированного (aOR, 0.6; 95%CI, 0.5-0.9) были независимым коррелятом с поиском лечения в клиниках среди МСМ Китая.
Заключение: Эта высокая распространённость ЗППП-инфекции в сочетании с низкой долей лиц, которые проявляют соответствующее поведение лечение-поиска, создают высокий риск растущей эпидемии ВИЧ-инфекции среди МСМ Китая. Модели, которые оказывают предпочтение лучшего скрининг для и образование о ЗППП должно быть срочно осуществлены, среди малообеспеченных с МСМ особенно.

Translated from English version into Russian by Hao-Qi Zhang.

Comportamiento de búsqueda de ayuda y barreras para el acceso a servicios de asistencia para enfermedades de transmisión sexual en hombres que tienen sexo con otros hombres en China: una encuesta transversal en múltiples centros

Jun-Jie Xu, Yan-Qiu Yu, Qing-Hai Hu, Hong-Jing Yan, Zhe Wang, Lin Lu, Ming-Hua Zhuang, Xi Chen, Ji-Hua Fu, Wei-Ming Tang, Wen-Qing Geng, Yong-Jun Jiang, Hong Shang

Resumen
Antecedentes: Las demoras en el tratamiento o el tratamiento inadecuado de enfermedades de transmisión sexual (ETS) aumenta el riesgo de adquisición de VIH y puede producir otros resultados perjudiciales. Sin embargo, son escasos los estudios sobre el comportamiento de búsqueda de ayuda para ETS y factores asociados en hombres que tienen sexo con otros hombres. Esta información es fundamental para la promoción del comportamiento de búsqueda de ayuda en las ETS y la disminución de la transmisión del VIH entre hombres que tienen sexo con otros hombres en China.
Métodos: Durante los años 2012 y 2013, se llevó a cabo un estudio transversal en múltiples centros en siete ciudades de China. Los participantes completaron una entrevista-cuestionario y dieron muestras de sangre, que luego fueron analizadas en búsqueda de anticuerpos contra el VIH, sífilis y el virus del herpes simple-2 (VHS-2). Los hombres que tenían sexo con otros hombres que resultaron positivos para sífilis/VHS-2 o que tuvieron síntomas obvios asociados con ETS dentro

de los últimos 12 meses, fueron definidos como hombres que tienen sexo con otros hombres con sospecha de infección por ETS.

Resultados: De los 4496 hombres que tienen sexo con otros hombres elegibles que completaron esta encuesta, 24,4% (1096/4496) fueron categorizados como hombres que tienen sexo con otros hombres con sospecha de ETS. 35,7% (391/1096) de estos hombres que tienen sexo con otros hombres con sospecha de infección por ETS buscaron ayuda en clínicas dentro de los últimos 12 meses. Entre los hombres que tienen sexo con otros hombres que no asistieron a clínicas de ETS para tratamiento, la prevalencia de sífilis y VHS-2 fue significativamente más elevada; la prevalencia del VIH y la incidencia dentro de esta subpoblación llegó hasta el 14,5% y 12,2/100 persona-años, respectivamente. El análisis de regresión logística multivariable indicó que el tener 7 a 12 de educación (comparado con ≤6 años; RP*a*, 2,5; 95%*IC*, 1,0-6,1), ≥13 años de educación (comparado con ≤6 años: RP*a*, 2,8; 95%*IC*, 1,2-7,0), ingresos mensuales >500 USD (comparado con ≤500 USD: RP*a*, 1,5; 95%*IC*, 1,1-2,1), síntomas obvios asociados a ETS dentro de los 12 últimos meses (RP*a*, 5,3; 95%*IC*, 3,7-7,5), estar infectado por VIH (RP*a*, 1,7; 95%*IC*, 1,1-2,6), estar actualmente infectado por sífilis (RP*a*, 0,6; 95%*IC*, 0,4-0,9) e infectado por VHS-2 (RP*a*, 0,6; 95%*IC*, 0,5-0,9) fueron correlatos independientes de la búsqueda de tratamiento para ETS en clínicas entre hombres que tienen sexo con otros hombres en China.

Conclusiones: La alta prevalencia de la infección por ETS sumada a una baja proporción de personas que exhiben comportamientos adecuados de búsqueda de ayuda crean un alto riesgo de una creciente epidemia de VIH entre hombres que tienen sexo con otros hombres en China. Deberían implementarse de inmediato modelos que prioricen un mejor tamizaje y educación sobre las ETS, en particular, entre hombres de bajos ingresos que tienen sexo con otros hombres.

Translated from English version into Spanish by Maria Alejandra Aguada, through

TRANSLATORS
WITHOUT BORDERS
